# Projections of IoT Applications in Colombia Using 5G Wireless Networks

**DOI:** 10.3390/s21217167

**Published:** 2021-10-28

**Authors:** Alexis Barrios-Ulloa, Dora Cama-Pinto, Johan Mardini-Bovea, Jorge Díaz-Martínez, Alejandro Cama-Pinto

**Affiliations:** 1 Department of Electronics Engineering, Faculty of Engineering, Universidad de Sucre, Sincelejo 700001, Colombia; alexis.barrios@unisucre.edu.co or; 2 Department of Computer Science and Electronics, Universidad de la Costa, Bicentennial Scolarship–Ministry of Sciences, Barranquilla 080002, Colombia; jdiaz5@cuc.edu.co; 3 Department of Computer Architecture and Technology, University of Granada, 18071 Granada, Spain; 4 Faculty of Engineering, Universidad del Atlántico, Barranquilla 081001, Colombia; johanmardini@uniatlantico.edu.co; 5 Department of Computer Science and Electronics, Universidad de la Costa, Barranquilla 080002, Colombia

**Keywords:** 5G, frequency bands, Colombia, IoT, radioelectric spectrum

## Abstract

Wireless technologies are increasingly relevant in different activities and lines of the economy, as well as in the daily life of people and companies. The advent of fifth generation networks (5G) implies a promising synergy with the Internet of Things (IoT), allowing for more automations in production processes and an increase in the efficiency of information transmission, managing to improve the efficiency in decision-making through tools such as big data and artificial intelligence. This article presents a description of the 5G implementation process in Colombia, as well as a revision of opportunities when combining with IoT in featured sectors of the departmental development plans, such as agriculture, tourism, health, the environment, and industry. Results shows that the startup of 5G in Colombia has been a slow process, but there are comparisons with similar procedures in other developed countries. Additionally, we present examples of 5G and IoT applications which can be promoted in Colombia, aimed at improving the quality of life of their habitants and promoting economic development.

## 1. Introduction

The demand of mobile data services is one of the factors that conditions the development of future wireless networks. The 2020 report of Ericsson indicates that the mobile data traffic in that year was 50 Exabytes (EB)/month, and it is estimated that it could be incremented to 150 EB/month in 2024 [1]. This increase is associated with a higher need of services by users and requirements of technologies such as Internet of Things (IoT), Wireless Sensor Networks (WSN), and Artificial Intelligence (IA). In order to meet demand, work began in 2012 on a Fifth Generation wireless technology (5G) [2].

The 5G network presents considerable improvements over fourth generation networks (4G), highlighting the increment of the transmission rate, ultra-reliability, less latency, and higher connectivity [3,4]. Some countries (Japan, South Korea, China, United States, Germany) finished test stages and began technology commercialization. Colombia has not been oblivious to this evolution, currently having 5G networks in pilot test processes before launching its service to the public. In 2020, the Ministry of Information and Communication Technologies (MinICT) issued a resolution, opening a process of granting permissions for the use of the radioelectric spectrum in different frequency bands, with the purpose of starting pilot tests which uses 5G mobile technologies [5]. Also, the ministry of ICT states that the 5G frequency bands’ concession stage will start before September 2022 [6].

### 1.1. IoT and 5G Networks

IoT is increasingly involved in different areas, including home automation, health, agriculture, industry, and logistic. The 5G networks exceed the limitations of 4G in terms of security, low latency, higher data transfer speed, and a higher quantity of connected devices on their communications, allowing for the possibility of a wide implementation of IoT applications [7,8]; many of which have low potency and latency requirements [9]. Therefore, different initiatives worldwide (for example, 4G America, IMT-2020, 5G Forum) have worked on the adoption and standardization of IoT enabled for 5G [10]. Also, the 3rd Generation Partnership Project (3GPP) has included IoT on their standards. For example, in June of 2020, an Industrial IoT (IIoT) work item was initiated and completed as a central element of Release 16, and is oriented to robotic applications and autonomous systems (RAS) [11].

In addition to the industrial field, it is possible to integrate IoT, through 5G, with other sectors, such as home, cities, and farms [9]. In Table 1, a general description of the typical requirements of IoT applications that the fifth generation networks should satisfy is presented.

However, despite the advantages of 5G IoT, its implementation will bring a series of challenges that need to be overcome, including scalability, network management, interoperability, and heterogeneity [12].

### 1.2. 5G Application Scenarios

**Figure 1:**
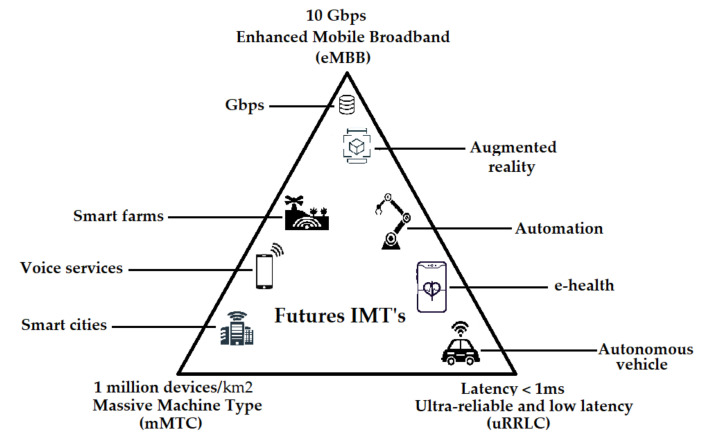
5G Application Scenarios. Source: [16,17].

Networks commercially operating with 5G are supported with the technology New Radio (NR), specified by the 3GPP in the Release 15, which is largely based on the recommendations given by the International Telecommunications Union (ITU) [13]. Some of the most important technical characteristics are the use of Orthogonal Frequency Division Multiplexing (OFDM) and the operation up to 52.5 GHz, through time division duplex (TDD) or frequency division duplex (FDD) [14]. In addition, Release 15 added new bands for 5G: 3300 MHz–4200 MHz, 3300 MHz–3800 MHz, 4400 MHz–5000 MHz, 24,250 MHz–27,500 MHz, and 37,000 MHz–40,000 MHz [15]. For the purpose of satisfying the future requirements of the market and especially of the industry, ITU divided 5G into three application scenarios [16]: (a) enhanced mobile broadband (eMBB), focused on a high transmission rate and targeting applications in busy indoor and outdoor environments, as well as a possible last-mile solution in areas where there is an absence of copper or fiber optic wired networks; (b) massive machine-to-machine communications (mMTC), specially designed to support a high density of equipment per square kilometer, such as IoT applications in smart environments, e.g., smart cities, smart farm, smart home, among others; and (c) ultra-reliable low latency communications (uRLLC) with lower transmission rates, intended to support industrial automation or applications requiring mobility, such as autonomous vehicles or patient monitoring. Figure 1 presents a summary of the main characteristics of the three categories.

Part of the 5G development is based on the fact that some applications will work in frequencies located in much higher bands than the ones used by the current 4G networks, as is the case with the so-called “high band” spectrum range between 24 GHz and 100 GHz [8]. To this end, the use of tools is required to minimize the problems associated with loss in propagation [18]. For this reason, the implementation of technologies of multiple massive transmission and reception (mMIMO) antennas that use spatial multiplexing to improve the channel capacity is considered, in addition to using large-scale antenna systems, and thus serving the growing number of users [14].

### 1.3. Goals and Motivation

- The main objective of this article is to show the progress for the deployment of the 5G network in Colombia, as well as the availability of the spectrum and the future needs of this resource according to the considerations established by regulatory agencies. In addition, an analysis of potential IoT applications in different sectors of the Colombian economy that could contribute to the development of the regions with the implementation of 5G is presented. The specific objectives of this article are:
- Associate IoT applications with 5G in the future demand of the main economic activities of each department and region in Colombia.
- Contextualize the current situation of 5G network deployment in Colombia.
- Relate the 5G frequency bands that will be implemented in Colombia with the most suitable IoT applications for the demands of economic activities in each department and region.

Our work will be a roadmap for future research in the development of IoT applications with 5G, according to the demand of the most relevant economic sectors in different parts of Colombia.

In this research, we present the demand for IoT applications that will be enhanced when working in combination with 5G networks in Colombia. For this purpose, a search was conducted in the Scopus database, selecting articles published as of 2017. We used search strings where the terms “5G”, “IoT”, “Colombia”, “spectrum”, “frequency bands”, “trends”, “challenges”, “agriculture”, “livestock”, “e-health”, “tourism”, “environment”, and “industry” were a part of the titles, abstracts, and keywords. Subsequently, we reviewed each of the documents, focusing on the issues raised, the results, and the conclusions. Additionally, we analyzed different documents from official regulatory organizations, manufacturers, operators, and governmental institutions, obtaining a more commercial vision of the future of 5G in Colombia.

The following criteria were considered in the final selection of the bibliography: a focus on IoT, a focus on IoT/5G, the relation of the subject matter with the Colombian context, the relevant problems of the Colombian economic and social sector, and government information. In total, we selected and used 193 references, of which 69 correspond to journal articles or conferences, 10 to private companies or organizations, 12 to regulators or foreign entities (external to Colombia), and 102 to Colombian public sector entities.

The structure of this article is as follows: Section 2 describes an analysis of the results and provides examples with the use of IoT and 5G in different sectors of Colombia. Finally, Section 3 shows the discussion and conclusions.

## 2. Results and Analysis

### 2.1. Projected Use of 5G Frequency Bands

Current saturation of the radioelectric spectrum has highlighted that, in different countries, the new auctions should principally be between 1 and 6 GHz. However, the growing need for a bandwidth that supports 5G services has brought into consideration portions of the spectrum that were previously not widely accessed; for example, the sub-GHz and the millimetric (mm-W). In Table 2, we highlight some examples of bands auctioned in some countries and their expectations about the allocation of 5G spectrum in the future.

In the Colombian case, to cover future needs and in harmony with the provisions of the 2019 World Radiocommunication Conference (WRC-19) [13], there is a need to arrange the spectrum in three (03) types of bands of frequency: (a) lower than 1GHz, (b) between 1 and 6 GHz, and (c) higher than 6 GHz. In this sense, the 5G plan developed by MinICT has identified a set of candidate frequencies to be used in future networks [26], which are presented in Table 3.

All of these bands have not been attributed to the mobile service provision according to the presented data on the National Table of Attribution of Frequency Bands [27], which will force entities to perform modifications in the future, or perform studies that allow for an evaluation of their compatibility with other already attributed services. Actually, there is not a formal definition of the 5G frequencies that will be assigned in Colombia; however, MinICT advances studies of viability about spectrum portions that could serve as IMT (International Mobile Telecommunications) bands, which should finalize in the fourth trimester of 2021 [28]. Also, in June 2020, MinICT invited the operators to manifest their interest to participate in processes of permission, obtaining radioelectric spectrum use for the provision of IMT services in the 700 MHZ, 1900 MHZ, 2500 MHz, and 3500 MHz bands [29].

Other bands that are considered by MinICT in the 5G Plan (31 GHz, 40 GHz, 71 GHz, and 81 GHz) were not identified by the WCR-19 for the provided 5G service. However, they could be a part of future spectrum auctions thanks to the developments of manufacturers and the needs of operators. Between the examples of this situation, there is the band of 28 GHz, not identified by the WCR-19 inside the set of destined frequencies to IMT, in which the 5G is framed. However, as it can be observed in Table 2, some countries have already auctioned in this spectrum portion, and others have manifested interest on doing so in the future.

### 2.2. Projected 5G Frequency Bands for Use in Colombia

In April 2020, MinICT authorized 5G pilot tests in Colombia in five (05) bands of the spectrum: 3.5–3.7 GHz, 24.25–27.5 GHz, 37–43.5 GHz, 45.5–47 GHz, and 47.2–48.2 GHz. Nevertheless, in agreement with the inform of assignation of the direction of the industry of Communications of MinICT, four of the network providers and telecommunication systems (NPTS) operating in Colombia submitted applications to perform tests in the band of 3500 MHz to 3600 MHz, while others with an interest submitted their application in bands from 3300 MHz to 3400 MHz and from 587 to 592 MHz [30]. In total, 52 entities (public and private) and 24 natural persons showed an interest in participating in this process for use in eight different types of activities in the next descending order of demand [17]: The first is the interest in using it in projects related to smart cities, followed by its use in applications in education, agricultural development, the entertainment industry, virtual reality, public safety, the health sector, and finally, in transportation.

Of the operators with participation in the mobile services in Colombia, three of them submitted applications to carry out pilot tests: Colombia Telecommunications, COMCEL Cellular Communication (Claro), and the Bogota Telecommunications Company. In those tests, the Claro company recorded speeds from 864 Mbps in the download link (DL) and 103 Mbps in the upload link (UL) [31]. The Movistar company, in alliance with the military hospital and with the MinICT endorsement, incorporated this technology to a telemedicine car with the purpose of providing services of interconsultations and an observation of procedures through video conferences. The results show that they reached rates of 1.62 Gbps in DL and 176 Mbps in UL [32]. Other values obtained by mobile telephone providers in Colombia, before the permissions granted in 2020 in indoor scenarios, reached speeds of 640 Mbps per cell and a spectral efficiency of 32 Mbps per MHz [33]. The Claro company in 2018 had speeds of 10 Gbps in the 28 GHz, and a latency lower than 1ms [34].

**Figure 2:**
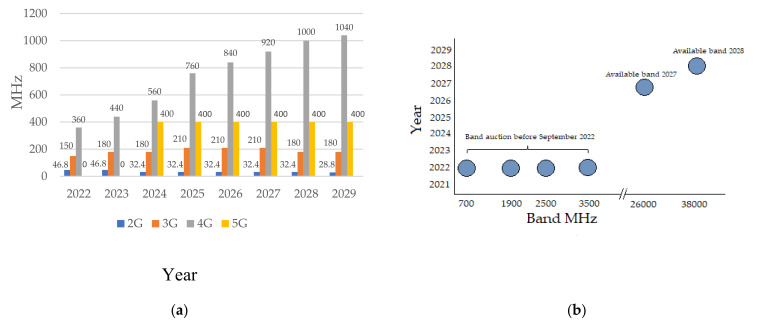
(**a**) Prospective spectrum demand for Colombia IMT, and (**b**) Band auctions projections. Source: ANE and [17].

Regarding the auctions of spectrum destined for 5G in Colombia, there is no set date, although there are some projections. The ANE has suggested that, with the entry of 5G, a spectral portion of 400MHz is necessary in the C band (3300–3700 MHz) in 2018, and close to 1172 MHz in the year 2024 [35]. Figure 2a,b shows a projection of ANE for the demand for mobile technology of its bandwidths in MHz until the year 2029, and the expectations for future spectrum auctions in the country.

Two scenarios have been put forward for 5G implementation from 4G-LTE: StandAlone (SA) and Non-StandAlone (NSA) [17]. Regarding SA, these are radio access architectures that do not use the existing 4G networks core, while in NSA, the 5G architecture is based on a 4G network core. In the Colombian case, it is expected that the first commercial networks will start in NSA mode, and that is why MinICT has been strengthening the 4G expansion process in Colombia, for which spectrum auction processes have already been carried out in the 700 MHz and 2500 MHz bands to expand mobile broadband coverage to 3658 locations located in rural areas. In addition, operators Tigo and Claro will execute the transition from 2G and 3G to 4G within four years, starting in 2021 [36,37]. Besides, in 2020, Colombia opened a consultation for expressions of interest in obtaining radio spectrum for the provision of IMT, of which 5G is a part. The bands destined for a future granting of permits are: 700 MHz, 1900 MHz, 2500 MHz, and 3500 MHz [29].

IoT is largely responsible for this increase in spectrum requirements in 5G, and this is demonstrated by the forecasts of different entities [38,39,40]. For example, CISCO, in its annual internet report, predicts that in the year 2023, the number of M2M (machine-to-machine) connections will be 14.7 billion, with an average of 1.8 connections per user worldwide [41]. In this report, they also present figures about the IoT applications with the highest number of connections, these being those related to the home. In addition, smart city and vehicle related applications will be faster growing.

Regarding IoT in Colombia, in accordance with the 5G Plan and in harmony with world trends, MinICT proposes possible uses in each of the 5G spectrum portions. Frequencies below 1 GHz will preferably be used for high-speed mobile broadband in urban, suburban, and rural areas, in order to serve the deployment of IoT services. Regarding the band between 1 and 6 GHz, it is the one with the greatest options to be used in the first commercial 5G implementations, and has 3G and 4G service assignments. As for the frequency band above 6 GHz (with or without a license), its use is proposed for ultra-fast speed connections.

A study presented by the General Directorate of Communications Networks, Contents, and Technology (an official entity of the European Community) indicated that approximately 19 GHz of shared spectrum could be required for IoT in the coming years [42]. The same report shows that there is not enough spectrum in the lower 1 GHz bands (low band) and between 1 to 6 GHz (mid-band) if you want to develop monitoring applications for highways, power supplies, and healthcare. It is important to highlight that they arrived at this result from three factors: (a) total number of devices per km2, (b) data rate of the devices, and (c) spectral efficiency. In terms of spectrum usage preferences in IoT, mobile operators prefer to implement IoT within licensed spectrum, while manufacturers lean towards implementation in unlicensed bands to avoid licensing costs [43].

### 2.3. Opportunities for Using IoT/5G Applications for Projects in Colombia

The National Development Plan (NDP) is an official document prepared by the National Planning Department of Colombia, which contains the strategic guidelines of each government policy [44]. It is a roadmap that details various aspects for improvement, projected for a period of four years. It is issued at the beginning of the mandate of each presidential government in order to ensure the continuous progress of the nation in different aspects, such as social, economic, environmental, and health. In this sense, one of the goals in the 2018–2022 four-year period is the increase in the number of internet connections and the digital transformation of society [45]. Our research is focused on the areas where the explosive growth of IoT applications is envisaged to work in conjunction with 5G wireless networks to become ideal solutions in different fields of national development.

**Figure 3:**
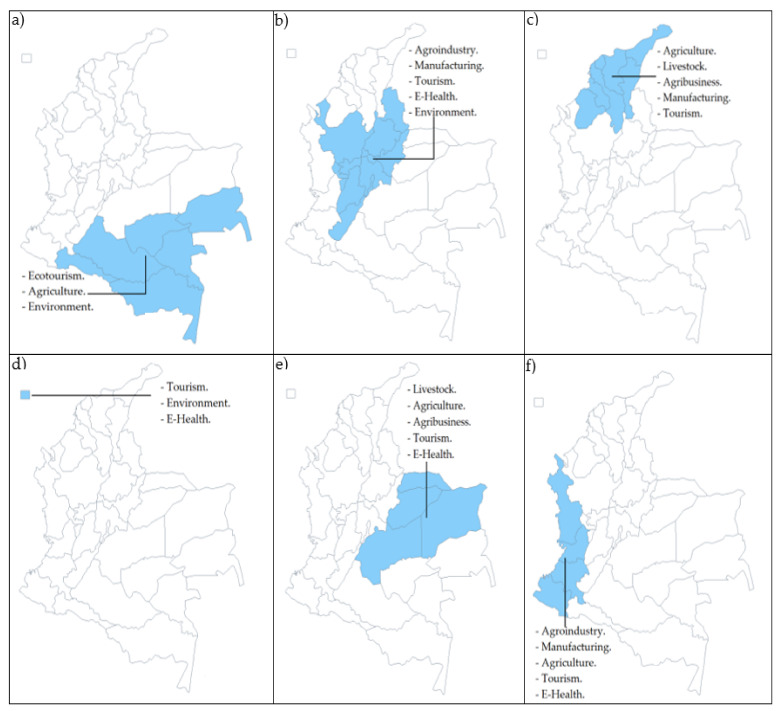
Potential demand for IoT with 5G in natural regions of Colombia according to DDPs: (**a**) Amazon, (**b**) Andean, (**c**) Caribbean, (**d**) Island, (**e**) Orinoco, and (**f**) Pacific.

Administratively, Colombia is made up of departments, districts, and municipalities in its political division with the largest geographical extension. However, historically it has also been subdivided into six (06) natural regions: Amazon, Andean, Caribbean, Island, Orinoco, and Pacific, which can be seen in Figure 3. Each of them is made up of different departments with their own geographical, social, and cultural characteristics that differentiate them from each other. Although there is an NDP, each department draws up its own government policy through a Departmental Development Plan (DDP), which is a planning instrument for a four-year period that allows governors to set the objectives and goals of their period, starting from its potentialities and shortcomings [46]. Through the DDPs, the aim is to strengthen different economic activities that help improve the quality of life of its citizens, and within this framework, IoT and 5G are promising tools that could help generate innovation and solutions to different problems. This section presents a summary of the economic activities in which each department has proposed its development efforts in the 2020–2023 period (see Table 4), and subsequently, the potentialities of IoT framed in 5G are analyzed for the advancement of the regions.

#### 2.3.1. Cattle Raising

Cattle raising is an activity carried out in a large part of the rural territory of Colombia, and it was one of the few economic sectors that showed growth in 2020 [126]. The PND promotes sustainable cattle raising and the development of agricultural production models with the support of technologies that increase their efficiency.

The proposed application possibilities of IoT focused on precision farming are varied. For example, monitoring the heat (when it is ready for mating) and the geographic location of the animals would allow for an increase of their reproductive efficiency, and protect against theft of cattle [127]. Diseases and health problems suffered by animals on farms cause a loss of productivity, and are a threat to public health [128]. In this sense, it would be possible to control animal diseases such as echinococcus [129] or tumors [130] through IoT management and monitoring systems. It will also be possible to help farmers in making decisions regarding livestock, thanks to AI techniques and machine learning (ML), among others [131]. Currently, there are no reports of a large number of IoT projects oriented to cattle farming in Colombia, although work has been done on the control of livestock mobility [132] or on the detection of animals in heat [133]. This utility of IoT will be enhanced with the improved capacity (bps) of 5G, thanks to increased bandwidth and spatial multiplexing, reducing network congestion and overload [127]. In addition, the reduction in energy consumption and the ability to connect a large number of devices make it possible for IoT to play an important role in the wide geographical scenarios where livestock farming is developed [131].

In this sense, 5G will reinforce the communication infrastructure of WSN, being the transport method to send data to the internet, preferably when using the sub-GHz band (700 MHz) in rural areas. Furthermore, and taking into account the high demand for wireless nodes deployed in large geographical areas required for smart farm environments, mMTC is the ideal option in a 5G scenario to serve the development purposes of this economic sector through ICT.

#### 2.3.2. Tourism

Before the start of the pandemic caused by the appearance of the SARS-CoV-2 virus (also known as COVID-19), tourism had achieved record figures in Colombia, reaching 4,515,932 non-resident foreign visitors, growing at a rate greater than the rest of the countries of the region [134]. Most Colombian departments consider tourism a key factor in their development, and for that they have a diverse offer. Although there are traditional tourist proposals for sun and beach (or coastal tourism) mainly in Bolívar, Magdalena, Sucre, and San Andrés—or historical tourism such as Cartagena and Mompox (in the department of Bolívar), Santa Marta (capital of Magdalena), or Popayán (capital of Cauca)—there are also many other alternatives such as adventure and ecological tourism in Chocó, Caquetá, Vichada, and Tolima with their jungles and natural reserves, while due to their biodiversity, the natural parks and the eastern plains are attractive in the departments of the Andean region and the Orinoco, respectively. In addition, the festivities and events also attract a large number of tourists each year, as in the case of Barranquilla (capital of the department of Atlántico) with its carnival, Medellín (capital of the department of Antioquia) with the Feria de las Flores, Pasto (Capital of the department of Nariño) with the Carnival of Blacks and Whites, or Cali (capital of the department of Valle del Cauca) with its fair. There are also health or medical tourism offers in some cities in Colombia, such as in the case of Barranquilla, Cali, Medellín, or Bucaramanga (capital of Santander). Thanks to the significant offer and investment in infrastructure and human training, these locations have been consolidated as suggested destinations to perform different types of interventions, mainly aesthetic and cardiology specialties [135,136,137].

The integration of IoT and 5G could provide great benefits to the tourism sector in these regions of Colombia, taking into account that despite its potential, many of these departments of tourism still do not represent a significant percentage of their economy, and therefore, there is a great growth opportunity. In this regard, and considering that the tourism industry requires a large amount of information and is increasingly dependent on ICT [138], it is necessary to collect information at all stages of a tourist trip or event, so that appropriate actions can be taken to satisfy customers and improve the competitiveness of this sector. The collection of information through IoT systems would help develop practical applications for the benefit of users such as guided tours, smart shopping, or efficient travel management [138,139], as well as to the monitoring of architectural works in historic buildings with at-risk infrastructure [140]. It would also allow for the implementation of algorithms for predicting user behavior as proposed in [141], or be complemented with big data tools so that tourists have a personalized experience and are able to make decisions about their trips [142,143]. It would also help to improve the experience and safety of travelers who make use of extended reality technologies through sensors, as in [144], where they used ultrasound transducers that perceive the surrounding environment, providing complementary information to augmented reality systems. The 5G network plans to offer small-cell and mm-Wave connectivity to tourists at any time and location [145]. So-called “green, ethical, and clean” tourism will best integrate IoT with 5G, boosting the use of virtual reality, augmented reality, and AI, thanks to the expected high speed and coverage [146].

In the Colombian context, virtual reality and augmented reality are the technologies of current interest in the development of prototypes. For example, in [147], they were used in the promotion of historical sites in Cartagena, or to improve the experience of tourists visiting tourist sites in Bucaramanga [148].

Considering the transmission of large amounts of data, as well as the efficient management of a high density of equipment in small areas due to the high number of tourists traveling for entertainment purposes, meeting in open spaces (beaches, festivals, fairs) or in enclosed areas (museums, historical buildings), and also for health tourism reasons, it is necessary to measure different variables to connect, monitor, and diagnose remotely [149]. Here, the 5G eMBB application scenario is the best option to support the communication needs and information volume in IoT applications in the tourism sector.

#### 2.3.3. Agriculture

All DDPs present the intention of strengthening the agricultural sector in each of the Colombian departments. Due to the geographical characteristics of each of them, different crops predominate that could benefit from the interaction of IoT, such as cassava in the Caribbean and Amazon region, coffee in the Andean region, sugarcane in the Pacific region, and rice in the Orinoco region. Despite the hydrological potential of Colombia and the quality of some of its lands for agriculture, this country is regularly affected by droughts that originate from natural phenomena such as the so-called “*Niño*”. In addition, there are many other factors, among which are the appearance of pests, bad practices in crops, and little technification. These factors have an impact on productivity and influence the cost of food, putting food sustainability at risk. For this reason, the Ministry of Agriculture has given recommendations to mitigate the effect of these events, including the monitoring of information and alerts issued by the Institute of Hydrology, Meteorology, and Environmental Studies (IDEAM); an alternate means of water supply; generating alternative mechanisms to shade crops and animals; planning irrigation shifts for access to water; constructing reservoirs for water storage; and generating plans for pest control [150].

IoT offers many options that could help farmers in managing their crops. One such option is the implementation of low-cost automated irrigation systems that use wireless technologies for the purpose of monitoring soil moisture and verifying its operation through IoT [151,152]. It is also attractive to develop control technologies for irrigation or fertigation systems, and to plan the administration of crops, which allows for an increase in the yield of crops and, at the same time, a reduction in environmental risks [153,154,155]. Another possibility is the monitoring of the crop or of climatological variables to which plantation managers can access remotely and through different platforms, allowing for efficient management [156,157].

With the speed of data transfer offered by 5G, agricultural operations have had a positive impact since 2017, helping to improve crop management through the use of drones and/or robots, the real-time monitoring of variables, and data analysis, among many others [158]. Another favorable aspect in this economic sector is the improvements introduced in 5G, by reducing IoT implementation costs and increasing its use to more efficiently integrate the agricultural cycle, i.e., from the sowing stage to the distribution of products [159].

Regarding the Colombian scenario, agriculture is possibly the economic and social sector where most research is being done in relation to IoT applications. For example, in [160,161], IoT was part of the set of tools used in crop irrigation management, while in [162] it improved production in a tomato field. These and other opportunities for implementation in agriculture will be possible with 5G, requiring a great effort from the public and private sectors to promote the implementation of new IoT projects, with the 5G mMTC scenario being the most appropriate because it supports many nodes deployed in large areas of crops. Currently, many rural areas in Colombia do not have access to cell phone networks or to the Internet as a complementary tool in their agricultural activities. To overcome this obstacle, the Colombian government has reached agreements with the main mobile telephone operators of the country, extending the cellular telephone network infrastructure to 3658 new rural locations before the end of 2024. This new technological panorama is favorable for projecting new applications that optimize the processes of agricultural activities. Otherwise, without this wireless coverage, the high costs of implementing IoT projects in agriculture would be low or nonexistent, due to the installation of the infrastructure [163,164].

#### 2.3.4. Health

The health problems of the different Colombian departments are diverse. In some departments, among which Norte de Santander, Caquetá, San Andrés y Providencia, Córdoba, and Chocó stand out, there are deficiencies in health services across several of their respective localities that surround them. In addition, due to the extension of their territories and the lack of adequate road infrastructure or geography, timely access to health professionals and medical centers is difficult. For example, in Chocó, a large part of the towns that make up this department are more than 7 h away from the municipal seats or the capital [112], while in Nariño, 40% of the total extension of the department does not count with road connection [113], which makes access to doctors, laboratories, or hospitals difficult. Another common health problem in Colombian departments are respiratory, circulatory, and heart diseases, which occur even in more developed departments such as Valle del Cauca, Antioquia, and Santander. Health systems based on IoT and 5G are a great opportunity to improve the quality of services and increase coverage, especially in remote areas of these regions where access to health services can be expensive and slow due to the great distances to travel. For example, the department of Antioquia in its PDD considers that the activities of telemedicine, prehospital telecare, and remote diagnosis, among others, can be effective in improving health care in remote locations [76].

Real-time monitoring applications using body area networks (WBAN) can become indispensable tools, monitoring vital signs of patients located in quarantine areas, in high-demand hospital centers, or in remote locations. The data can then be sent to IoT devices, which then forward them to the treating physician or diagnostic systems using the 5G network [165,166,167,168]. Through IoT, it would also be possible to monitor patients who have travelled hundreds of kilometers to hospital centers, allowing health care workers to know vital parameters such as heartbeat, temperature, or respiratory rate in advance, making possible the provision of an efficient service with less loss of time. Some resources that are a part of an intelligent hospital infrastructure and that will dominate the health scenario in the future area include telecare assets, networked medical devices, networks, and sensors distributed in buildings [165].

IoT/5G deployment is currently contributing to the rise of healthcare applications because they leverage higher availability, high scalability, and low network latency [169]. In addition, multi-access perimeter computing nodes in 5G provide sufficient compute and storage capacities at the edge of the network [170].

Some prototypes of IoT applications in health care have already been implemented in Colombia for the remote monitoring of patients, as in [171], where large data sets were obtained through sensors to perform predictive analysis among a group of people with hypertension problems, and thus designing prevention campaigns; or in [172], oriented to the surveillance of this same disease. Other projects have been oriented to the remote monitoring of patients in hospital centers [173], or even to self-monitoring, in an attempt to avoid traveling to hospitals or clinics [174].

Critical IoT applications in the health sector will be potentiated with the advantages offered by 5G networks because they demand high reliability and low communication latency, being the uRLLC scenario appropriate for the sensitive registration of vital signs information collected by devices used for patients [165,175,176,177].

#### 2.3.5. Industry

Table 4 shows that manufacturing-type industries predominate in Colombia, mainly in departments with greater economic development such as Antioquia, Atlántico, Cundinamarca, Santander, and Valle del Cauca. The modern industry increasingly requires low latency, high determinism, high bandwidth, and high resilience computing and communication; characteristics necessary to implement fast, intelligent, and autonomous decision-making [178]. In this sense, IoT and 5G are considered technological tools of great potential, because industry 4.0 is increasingly incorporating wireless connections in the manufacturing and logistics of its processes [179]. In addition, an increase is expected in the number of industrial wireless sensors that collect information on environmental conditions and the processes carried out in the plants, allowing for self-diagnosis activities to be carried out for the maintenance and operation of the equipment [180]. There are also opportunities with industrial production robots that can be controlled in the cloud [178], for which 5G will be key in the timely exchange of information through high-speed connections [180].

The positive impact of 5G features on industry offers improved real-time production monitoring or information about the status of a piece of equipment or its manufacturing process [181]. Some projections indicate that the digitalization of the manufacturing industry enabled by 5G will offer new production opportunities, increasing global manufacturing revenues from USD 52 billion in 2022 to USD 233 billion in 2026 [182].

In Colombia, the Superintendence of Industry and Commerce presented the main aspects to implement IoT in the industrial sector, specifically in logistics, inventory management, and smart factories. It also identified a patent application by a Colombian company on the issue of tracking for the control and traceability of the position and fixed or moving location of tangible objects or merchandise by means of radiofrequency and image recognition [183]. Because industrial services are classified as critical, it is convenient to make use of the uRLLC scenario, so that the probability of failure at low latency is reduced [184,185].

#### 2.3.6. Environment

Deforestation is the predominant environmental problem in the regions that make up the Colombian territory. Figures from the Ministry of Environment and Sustainable Development (Minambiente) indicate that in 2020, 171,685 hectares of forest were lost in Colombia as a result of deforestation [186].

Some actions have been carried out with the purpose of finding solutions through IoT that allow reducing deforestation levels with the implementation of technologies that detect illegal logging and the commercialization of wood. Amazon Web Services (AWS), in collaboration with the Jorge Tadeo Lozano University and the United States Embassy, sponsored an event in 2019 where they faced challenges to control the production chain, and combatted illegal logging and deforestation. One of the solutions proposed was based on IoT, and consisted of tracking the record from the moment of extraction to the delivery to manufacturers through devices installed in trucks [187]. Also, in [188], the advantage of IoT aimed at preventing illegal deforestation is demonstrated, integrating inclination and sound sensors to detect the poaching of trees. Regarding forest fires, DDPs have identified them as one of the main environmental problems in Colombia, causing serious damage to the biodiversity of the areas where they occur, as well as deterioration in air quality in cities [189]. IoT can also be a part of the solution to this phenomenon; more specifically, its application in this field is a topic widely researched and reported in the literature, and, therefore, it could serve as an example of implementation to present solutions in Colombia. For example, in [190,191,192], they used IoT devices with meteorological sensors (rain, temperature, humidity, wind speed, CO, CO_2_) for the detection and prediction of forest fires, respectively, making timely decisions that help to preserve forests and jungles.

There is also research on air pollution and environmental temperature and humidity monitoring that prefers 5G technology to transmit data over the cloud rather than Zigbee, Bluetooth, and Wi-Fi, because of its high communication range and low power consumption [193].

Regarding 5G, a wide use of this technology is not reported in applications related to the topic discussed in this item. However, if these networks are deployed in the future, it would be possible to develop similar projects in Colombia, especially with the use of bands below 1 GHz (700 MHz) that have been considered for use in rural areas, because they have lower attenuation with respect to other higher frequency bands due to the characteristic of its wavelength. Also, the mMTC 5G scenario is the best option for implementation in IoT applications that monitor environmental variables in the aforementioned areas of study and research.

**Figure 4:**
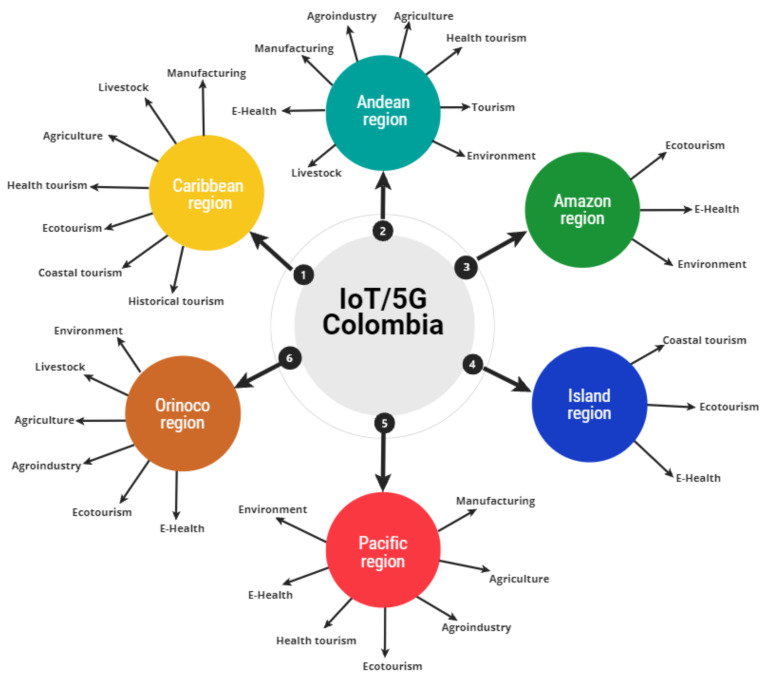
IoT/5G applications potential summary in natural regions of Colombia.

The application possibilities of IoT based on 5G are wide and generate a high expectation of development in the Colombian economy and society. Moreover, sectors such as education, security, sports, and transportation, among others, could also benefit from the joint implementation of these two technological tools. Figure 4 summarizes the comparative analysis of the possibility of applications for each natural region of Colombia.

## 3. Discussion

It is evident that 5G represents an important evolution in the development of wireless networks passing from focusing on users, to machines, to the industry, and through the cloud, within the Internet of Things. Moreover, changes are observed in the spectrum management with respect to what was done by most nations until the deployment of fourth generation networks, currently considering the use of bands above 6 GHz, which brings more bandwidth, but also creates challenges in relation to the propagation of radio waves. However, we believe that all of the advantages offered by 5G will not be available before September 2022. This is due to the fact that, since 4G is the base that initially supports it, the latter is still expanding its deployment in most of the rural areas of the Colombian territory, promoted by bilateral agreements between the state and private companies. Therefore, it will be necessary to increase regulatory policies that allow operators to expand their coverage, as well as incentives for users to acquire the necessary services and equipment to implement IoT in different areas of the economy; for example, the current tax exemption for the purchase of smartphones that are below approximately USD 185.

This review shows that there is a wide range of tourism in parks and nature reserves, which, together with agricultural activities, deforestation, and major health issues, are more relevant in rural scenarios. Consequently, the short- and medium-term use of IoT through 5G as technological tools to help solve challenges in health, environment, agribusiness, and eco-tourism, among others, will depend on different projections and agreements between the state and private enterprise, highlighting:

- early execution of spectrum auction processes;
- incentives for operators to deploy infrastructure in areas where there is no high purchasing power by the population, and little opportunity for rapid return on investment;
- rapid regulation for spectrum below 1 GHz to be auctioned and promptly used in rural areas, to overcome propagation problems and the number of antennas that would have to be installed compared to higher frequency bands; and
- implementation of applications in 5G mMTC and eMBB scenarios to satisfy the high density of equipment per square kilometer, as well as high transmission rate requirements.

As for industrial activities, the results of health tourism and events such as trade fairs and congresses are mainly in large cities. As a result, the following should be considered:

- The spectrum solution to serve IoT applications in these cases should be oriented to bands above 1 GHz.
- In cases of applications that demand a high data rate, such as virtual reality and big data for tourism in major urban centers, and machine learning in the industrial sector, eMBB is the scenario that best meets this demand, increasing its channel capacity in the future with the use of millimeter bands.
- uRLLC is the 5G scenario that best meets IoT applications in e-health in hospitals, due to its low latency and better performance in highly mobile equipment.

In the Colombian case, it has been officially established that the first commercial 5G network will be available before the end of August 2022, with progress being made by MinICT in pilot tests and technical regulations.

Finally, the use of IoT applications with 5G described throughout the document is an opportunity to increase the economic and social wealth in Colombia, making viable the execution of future projects associated with the fundamental demands in different geographical scenarios throughout the Colombian territory.

## Figures and Tables

**Table 1:** General description of features/typical IoT application requirements.

Application	Aplication Domain	Tolerable Delay	Update Frecuency	Data Rate
Structural health	Smart city	30 min	10 min	Low
Waste management	Smart city	30 min	1 h	Low
Video Surveillance	Smart city	Seconds	Real time	High
Air Quality Monitoring	Smart home	5 min	30 min	Low
Monitoring and supervision	Industrial	Seconds or ms	Seconds	Low
Closed loop control	Industrial	Milliseconds	Milliseconds	Low
Interlocking and control	Industrial	Milliseconds	Milliseconds	Low
Patient’s healthcare delivery and monitoring	Healthcare	Low (seconds)	1 Report per hour day	High
Real-time emergency response and remote diagnostics	Healthcare	Low (seconds)	Requires Ad-hoc emergency communication	High

**Table 2:** Examples of auctioned bands for 5G service provision. Source: own elaboration.

Country	Bands Auctioned	Expectation	References
USA	3.7 GHz, 24 GHz, 28 GHz, 37 GHz, 39 GHz, 47 GHz	Auction the 2.5 GHz and 3.45 GHz bands	[19,20]
Spain	3.6 GHz–3.8 GHz	Auction the 700 MHz and 26 GHz bands	[21]
Greece	700 MHz, 2 GHz, 3.4–3.8 GHz, 26 GHz	Public inquiries for the 1.5 GHz, 2.1 GHz, 2.3 GHz bands	[22]
Sweden	2.3 GHz, 3.5 GHz	Have the band from 3.72 GHZ to 3.8 GHz	[23]
United Kingdom	Not assigned	Auction 700 MHz and 3.6 to 3.8 GHz bands	[24]
Japan	3.7 GHz, 4.5 GHz, 28 GHz	Assignment of the 28.3 GHz and 29.1 GHz bands	[25]

**Table 3:** General description of characteristics/typical requirements of IoT applications. Own elaboration based on information from MinICT and the National Spectrum Agency (ANE) and [17].

Band Type	Bands	Current Usage	Advantages	Disadvantages
Less than 1 GHz	614–698 MHz	Broadcasting and mobile service	Greater coverage than frequencies above 1 GHz	Lower channel capacity (bps) than frequencies above 1 GHz
	698–806 MHz	Mobile service		
Between 1 and 6 GHz	3.3–3.4 GHz	Mobile service	Balance between coverage and capacity	Saturated spectrum for mobile and wireless services
	3.4–3.6	Fixed service, mobile service, and satellite service		
	3.6–3.7 GHz	Fixed service, mobile service, and satellite service		
Over 6 GHz	24.25–27.5 GHz	Fixed service, mobile service, and radio navigation	Channel availability increased and spectrum saturation reduced	Lower coverage than other bands aforementioned
	26.5–29.5 GHz	Fixed service, mobile service, and satellite service		
	31.8–33.4 GHz	Fixed service, radio navigation, and space research		
	37–40.5 GHz	Fixed service, mobile service, and satellite service		
	40.5–42.5 GHz	Fixed service, mobile service, satellite service, and broadcasting		
	42.5–43.5 GHz	Fixed service, mobile service, satellite service, and radio astronomy		
	45.5–47 GHz	Mobile service, satellite service, and radio navigation		
	47–47.2 GHz	Amateur		
	47.2–50.2 GHz	Fixed service, mobile service, and satellite service		
	50.4–52.6 GHz	Fixed service, mobile service, and satellite service		
	66–71 GHz	Fixed service, mobile service, satellite service, and radio navigation		
	71–76 GHz	Fixed service, mobile service, satellite service, and broadcasting		
	81–86 GHz	Fixed service, mobile service, satellite service, and radio astronomy		

**Table 4:** Economic activities, problematics, and potentialities of Colombia departments.

The Caribbean Region							
Department	Agriculture	Tourism	Cattle Raising	Health	Industry	Environmental	References
Atlántico	Corn, yucca, mango, citrus, pigeon pea, sorghum, melon	Health tourism, Barranquilla Carnival, Barranquilla Zoo, Santa Verónica beaches	Fish farming, poultry farming, cattle, pigs, sheep	Mortality from malignant tumors, mortality from ischemic heart diseases	Manufacturing, cement, agribusiness, chemical, port, electricity generation	Erosion, contamination of water sources, floods, forest fires	[47,48,49,50,51,52,53,54,55,56,57]
Guajira	Corn, coffee, yucca, rice, banana, bean, oil palm	Natural parks	Aquaculture, goats, sheep, equine cattle, fish farming	Communicable diseases, diseases of the circulatory system	Mining, port electricity generation, salinera	Desertification	[50,54,55,56,58,59,60,61]
Bolívar	Corn, oil palm, yucca, rice, yam, banana, cocoa, avocado	Hotels, historic center of Cartagena, historic center of Mompox, fairs and events, beaches, Totumo Volcano, Salinas de Galerazamba	Fish farming, poultry farming, beekeeping, cattle, equine cattle, sheep cattle	Ischemic diseases, diseases of the circulatory system, hypertension, cardio-cerebrovascular diseases	Agribusiness, manufacturing, petrochemical mining, port, electricity generation	Greenhouse gas (GHG) emissions, impacts of climate change in coastal areas, natural phenomena	[50,52,53,54,55,56,57,61,62,63,64,65]
Cesar	Palm oil, yucca, banana, rice, corn, pin, melon	Vallenato festival, ecotourism and ethno-tourism in the Sierra Nevada de Santa Marta and Serranía del Perijá, Ciénaga De la Zapatosa	Sheep, goat, cattle, fish farming, poultry	Maternal mortality, infant mortality from Acute respiratory infection (ARI), infant mortality from acute diarrheal disease (ADD)	Mining, agribusiness	Impact by climate change, impact by natural disasters, deforestation	[50,52,53,54,66,67,68]
Córdoba	Corn, banana, yucca, rice, yam, cotton, oil palm, cocoa	Ciénagas, Montería cattle fair, joint festival in San Pelayo natural parks, Gulf of Morrosquillo beaches	Cattle, pigs, equine cattle, sheep, goats, poultry, aquaculture, fish farming, beekeeping	High operational costs due to geographic conditions, ADD, obesity, diabetes, high blood pressure	Agribusiness, mining, electricity generation	Air pollution from mining exploitation, GHG emissions, deforestation, illegal hunting	[50,51,52,53,54,55,61,65,69,70]
Magdalena	Palm oil, corn, yucca, banana, coffee, citrus, mango, plantain	Hotels, Tayrona Park, Sierra Nevada de Santa Marta National Natural Park, Santa Marta and El Rodadero beaches, ecotourism	Marine fishing, cattle, sheep, poultry, fish farming	ARI, ADD, genitourinary diseases, cardiovascular diseases	Manufacturing, port industry	Deforestation, inappropriate land use	[50,52,53,54,60,71,72,73]
Sucre	Rice, yucca, corn, yam, banana, oil palm, pin	Gulf of Morrosquillo Beaches, January 20 festivities in Sincelejo, San Bernardo Islands Archipelago	Cattle, equine cattle, pigs, poultry, fish farming, beekeeping	ADD, ARI, dengue	Agroindustry, mining, cement industry, port industry	Illegal hunting, over exploitation of water sources, forest fires, deforestation, soil deterioration, pesticide contamination	[50,51,56,61,65,74,75]
**Andean Region**							
**Department**	**Agriculture**	**Tourism**	**Cattle raising**	**Health**	**Industry**	**Environmental**	**References**
Antioquia	Coffee, banana, cane, plantain, cocoa, corn, rice, flowers	Flower Festival, hotels, ecotourism, cultural tourism, health tourism	Swine, cattle, equine cattle, sheep, goats, poultry, fish farming, beekeeping	Cardiovascular diseases, respiratory diseases, hypertension, infrastructure problems	Manufacturing industry, agribusiness, mining, Center for the Fourth Industrial Revolution, electricity generation, port industry	GHG emissions, climate change risks, deforestation, illegal hunting	[50,51,52,54,55,57,61,65,76,77,78]
Boyacá	Potato, vegetables, cocoa, fruit trees, panelera cane, quinoa, cereals	Villa de Leyva, Chicamocha Canyon, Tunja, El Cocuy National Natural Park, Tota Lake.	Cattle, sheep-goat, beekeeping, poultry, pig and fish farming.	Diseases of the circulatory system, neoplasms	Agribusiness, mining, manufacturing industry, electricity generation	Deforestation, risks due to climate change	[50,52,53,54,57,65,79,80]
Caldas	Coffee, banana, panelera cane, avocado, citrus, cocoa, sugarcane	Coffee cultural landscape, avitourism, nature tourism, hot springs	Cattle, swine, fish farming, poultry farming,	Diseases of the circulatory system, diabetes mellitus	Agribusiness, metalworking industry, manufacturing industry, textile industry, electricity generation	Soil loss due to various anthropic activities, water bodies affected by discharges. deforestation	[50,51,52,53,55,57,81,82]
Cundinamarca	Potato, carrot, tomato, onion, lettuce, Corn, banana, sugar cane, flowers	Hotels, ecotourism, natural parks, forest reserves.	Cattle farming, sheep farming, goat farming, equine farming, pig farming, poultry farming, fish farming, beekeeping	Lack of insurance and access to health services in the municipalities	Manufacturing industry, agribusiness, chemical industry, electricity generation	Degradation of water reserve areas, risks due to climate change, forest fires, contamination of water sources	[50,51,52,53,54,55,57,61,65,77,83]
Huila	coffee, rice, banana, beans, corn, sugar cane, cocoa, yucca	Archaeological tourism in San Agustín, nature tourism in the Tatacoa desert, ecotourism	Cattle, swine, fish farming, poultry farming, beekeeping	Diseases of the circulatory system, neoplasms, ARI in children under 5 years of age, ADD	Agribusiness, mining, electricity generation	Impacts of climate change, contamination of water sources, loss of strategic ecosystems	[50,51,52,53,55,65,84,85]
Norte de Santander	Coffee, cocoa, oil palm, sugar cane, banana, avocado, rice, beans	Ecotourism, Villa del Rosario	Cattle, equine cattle, pigs, sheep, goats, poultry	Access to health in rural areas is limited, leprosy, malaria, dengue	Agribusiness, manufacturing industry, electricity generation	Pollution of water sources, deforestation	[50,51,53,54,55,61,86,87]
Quindío	Banana, coffee, citrus, avocado, banana	Parque del Café, coffee cultural landscape, ecotourism	Poultry, swine, cattle	They need to strengthen the hospital network	Agroindustry	Deforestation	[50,51,52,88,89,90]
Risaralda	Coffee, banana, avocado, sugarcane, corn, beans, tomato, onion and various vegetables	Coffee cultural landscape, natural parks, ecotourism, hot springs of Santa Rosa and San Vicente	Cattle, pig farming, fish farming, poultry farming	Diseases of the circulatory system, neoplasms, diseases of the respiratory system	Agribusiness, manufacturing industry,	Deforestation, contamination of water sources, loss of soil, air pollution, illegal hunting	[51,52,53,57,91,92]
Santander	Palm oil, cocoa, coffee, sugarcane, citrus, banana, rubber, pineapple, Yucca	Barichara, San Gil, Chicamocha National Park, natural parks, ecotourism, health tourism	Cattle, pig, equine, goat, sheep, poultry, fish farming	Heart disease and neoplasms are common causes of death. Public health strategies that stimulate sport will be worked on	Mining, oil, petrochemical, agribusiness, manufacturing, electricity generation, port	Deforestation, contamination of water sources	[50,51,52,53,54,55,56,57,61,93,94,95]
Tolima	Coffee, rice, corn, banana, beans, sugar cane, avocado, cocoa, sugar cane, mango	Ecotourism, adventure tourism and historical tourism	Bovine, equine, ovine, pig and poultry farming, fish farming	ARI, ADD, syphilis, diseases of the circulatory system	Manufacturing industries, agribusiness, mining, electricity generation	Risks due to climate change, contamination of water sources, deforestation	[50,51,52,53,54,55,61,96,97,98]
**Amazon Region**							
**Department**	**Agriculture**	**Tourism**	**Cattle raising**	**Health**	**Industry**	**Environmental**	**References**
Amazonas	Yucca, banana, various fruit trees	Flor de Loto Nature Reserve, Los Micos Island, indigenous communities, Lagos de Tarapoto	Swine, poultry, fish farming	Leptospirosis, diabetes, high blood pressure	manufacture	Deforestation, risks due to climate change	[52,99,100]
Caquetá	Banana, yucca, cocoa, rubber, coffee, cane, rice, corn	Ecotourism, adventure tourism, ethno-tourism	Cattle, swine, poultry, sheep	Lack of access to services, cardiovascular diseases, ARI	Agroindustry	Low quality of water for human consumption, contamination by chemical substances	[50,51,53,54,101]
Guainía	Banana, yucca, corn, cocoa	Ecotourism, Puinawual Natural Reserve, Cerros de Manicure	Swine, poultry, fishing	Tuberculosis, ARI, ischemic heart disease	Mining	Deforestation, effects of climate change	[51,52,102]
Guaviare	Corn, banana, yucca, rice, rubber, cane, cocoa	Ecotourism, Serranía de Chiribiquete Natural Park	Cattle, equine cattle, poultry	Lack of access to services, diabetes,	Mining, manufacturing industries	Deforestation	[52,61,103,104,105]
Putumayo	Yucca, corn, cacao, cane, chontaduro, pepper, cacao, banana	Ecotourism	Poultry, cattle, equine cattle, pig farming, fish farming	Diseases of the circulatory system, ARI, HIV, intestinal infectious diseases	Mining, agribusiness	Deforestation, floods	[50,51,52,53,61,106,107]
Vaupés	Yucca, cocoa	Ecotourism, ethno-tourism, nature reserves	Aquaculture	Lack of access to services, diseases of the circulatory system	Mining	Deforestation, forest fires	[108]
**Island Region**							
**Department**	**Agriculture**	**Tourism**	**Cattle raising**	**Health**	**Industry**	**Environmental**	**References**
San Andrés, Providencia y Santa Catalina	Coconut, yam, banana, yucca, corn	Hotels, beaches, ecotourism	Fishing, pigs, poultry	Obsolescence of infrastructure, diseases of the	Manufacturing, port	Effects of climate change, pollution of water sources,	[52,56,109]
				circulatory system, neoplasms		pollution of marine ecosystems	
**Pacific Region**							
**Department**	**Agriculture**	**Tourism**	**Cattle raising**	**Health**	**Industry**	**Environmental**	**References**
Cauca	Sugar cane, coffee, banana, sugar cane, corn, yucca	Holy week Popayán	Cattle, equine cattle, sheep, pig farming, poultry farming, fish farming, beekeeping	ARI, ADD	Agribusiness, manufacturing, electricity generation	Deforestation.	[51,52,53,54,55,57,61,63,110,111]
Chocó	Banana, corn, rice, cocoa and coconut.	Virgin beaches, ecotourism, adventure tourism.	Cattle, poultry farming, fish farming.	Lack of access to services.	Gold mining	Deforestation and water quality.	[50,52,53,112]
Nariño	Coffee, potato, banana, cocoa, oil palm, sugar cane, pea, corn, coconut, beans	Black and white carnival, Our Lady of Las Lajas Sanctuary, Doña Juana Volcanic Complex Natural Park	Cattle, equine cattle, sheep, marine fishing, fish farming, pig farming, poultry farming	Child mortality	Mining, port	Deforestation	[50,51,52,53,54,55,61,113,114,115]
Valle del Cauca	Sugar cane, coffee, banana, corn, sugar cane, citrus, plantain, rice, pineapple, avocado	Cali Fair, health tourism.	Cattle, poultry, swine, fish farming, beekeeping	Infant mortality, violence.	Agribusiness, manufacturing, rubber, port, chemical, electricity generation	Deforestation	[50,51,52,53,55,56,57,65,116,117]
**Orinoco Region**							
**Department**	**Agriculture**	**Tourism**	**Cattle raising**	**Health**	**Industry**	**Environmental**	**References**
Arauca	Banana, cocoa, rice, corn, yucca.	Natural parks, perpetual snow.	Cattle, sheep, poultry, fish farming	Deficiency in access to health services.	Agroindustry	Deforestation and disaster risk.	[50,52,53,54,118,119]
Casanare	Rice, oil palm, coffee, corn, banana, Yucca	Eastern plains	Cattle, equine cattle, sheep, pig farming, poultry farming, fish farming	Deficiency in access to health services.	Agribusiness, mining	Deforestation.	[50,51,52,53,54,61,120,121]
Meta	Palm oil, corn, sugar cane, rice, soy, banana.	Natural parks, caño crystals.	Fish farming, beekeeping, cattle, sheep, goats	Deficiency in access to health services.	Oil, electricity generation	Deforestation, sewage deficiencies.	[50,52,53,61,122,123]
Vichada	Soy, corn, oil palm, cashew, yucca, rice, rubber, wood	Ecotourism, sport fishing, adventure tourism.	Bovine and buffalo livestock	Malnutrition	Agroindustry (forestry). Resin distillation	Deforestation	[50,124,125]
